# Impact of COPD Exacerbations on Osteoporosis Assessed by Chest CT Scan

**DOI:** 10.3109/15412555.2011.650243

**Published:** 2012-02-23

**Authors:** Hirofumi Kiyokawa, Shigeo Muro, Tsuyoshi Oguma, Susumu Sato, Naoya Tanabe, Tamaki Takahashi, Megumi Kudo, Daisuke Kinose, Hiroshi Kondoh, Takeshi Kubo, Yuma Hoshino, Emiko Ogawa, Toyohiro Hirai, Michiaki Mishima

**Affiliations:** 1 Departments of Respiratory Medicine, Kyoto University, Kyoto, Japan; 2 Geriatric Medicine, Graduate School of Medicine, Kyoto University, Kyoto, Japan; 3 Department of Diagnostic Imaging and Nuclear Medicine, Kyoto University, Kyoto, Japan

**Keywords:** Exacerbation, Osteoporosis, Bone mineral density, Chronic obstmetive pulmonary disease, Emphysema

## Abstract

*Background:* COPD pathology involves not only the lungs but also extrapulmonary abnormalities. Osteoporosis is one of the most important abnormalities because it may cause vertebral compression fractures and deteriorate pulmonary function. COPD patients have many risk factors for osteoporosis, such as low BMI, decreased activity, systemic inflammation, and use of corticosteroids. Some of these factors have been shown to deteriorate with COPD exacerbations. We previously demonstrated the correlation between emphysema and osteoporosis and between emphysema progression and COPD exacerbations. Thus, the hypothesis that exacerbation causes osteoporosis progression in COPD patients was investigated. *Methods:* Forty-two COPD patients not on osteoporosis treatment for over 2 years were recruited. During follow-up, exacerbations had been prospectively recorded. Thoracic vertebral bone mineral density (BMD) was measured using chest CT, and the annual change in BMD was calculated. The change was compared between patients with and without a history of exacerbations. *Results:* The decrease in thoracic vertebral BMD was greater in patients with than in those without a history of exacerbations (median ABMD mg/ml year: −3.78 versus −0.30, p = 0.02). Moreover, multivariate regression analysis showed that exacerbations and baseline Pa0_2_ were independent predictors of the BMD decrease (R^2^ = 0.20, p = 0.007, and R^2^ = 0.09, p = 0.03, respectively) after adjustment for baseline age, smoking status, and airflow limitation. Conclusions: This is the first longitudinal study to demonstrate that COPD exacerbations are independently associated with osteoporosis progression. Osteoporosis progression should be evaluated in COPD patients, especially in those with a history of frequent exacerbations.

## Introduction

The pathology of COPD involves not only the lungs but also extrapulmonary abnormalities such as diabetes, cachexia, skeletal muscle wasting, and anemia (1). Of these, osteoporosis is one of the most important abnormalities because it may cause vertebral compression fractures (VCF) that can deteriorate pulmonary function. It has been reported that the prevalence of VCF in COPD patients is high (24% to 63%) (2), and one compression fracture in the thoracic vertebra results in a 9% decrease in vital capacity and impairment in FEV_1_ in COPD patients (3,4).

The common features in COPD patients, such as low body mass index (BMI), history of smoking, age, inactivity, systemic inflammation, and the use of systemic corticosteroids, are important risk factors for osteoporosis (3). Moreover, we previously reported that severity of emphysema was closely related to loss of vertebral bone mineral density (BMD) assessed by chest computed tomography (CT) scan (5), and our findings were subsequently confirmed by other investigators using conventional osteoporosis indices (Dual-emission X-ray absorptiometry) (6). These findings suggest that COPD pathology is closely linked to osteoporosis.

Exacerbation is an important issue in COPD because it has negative impacts on FEV_1_ (7,8), health status (9), survival (10,11), the BODE index (12), and socioeco-nomic costs (13). Exacerbations also cause deterioration in several risk factors for osteoporosis, such as inactivity (12) and systemic inflammation (14), and the use of systemic corticosteroids during exacerbations may accelerate BMD loss (11). Moreover, we recently reported that exacerbations accelerated emphysematous change (15). Thus, we speculated that exacerbations may accelerate not only parenchymal destruction but also BMD loss. However, to the best of our knowledge, no longitudinal study has examined the relationship between COPD exacerbations and osteoporosis progression.

Using chest CT scan images, BMD of the thoracic vertebrae and emphysematous changes can be evaluated simultaneously (16,17). Moreover, BMD of the vertebra assessed by chest CT scan is better than that assessed by dual X-ray absorption to predict vertebral VCFs (18). In the present study, the longitudinal impact of exacerbations on thoracic vertebral BMD was explored using chest CT scan data.

## Methods

### Subjects

This is a part of prospective observational study investigating COPD exacerbation (12,15,19). The patient inclusion criteria were: (1) COPD diagnosed according to the criteria of the Global Initiative for Chronic Obstructive Lung Disease (1); and (2) the patient agreed to prospec-tively record exacerbations. The exclusion criteria were: (1) history of respiratory diseases other than COPD; (2) occurrence of malignancy within the previous 5 years; (3) history of bone disease; (4) receiving osteoporosis treatment such as bisphosphonates and alfacalcidol (vitamin D analogue); (5) currently receiving oral systemic corti-costeroid therapy; (6) receiving rehabilitation during the study; and (7) receiving home-oxygen therapy.

**Figure 1:**
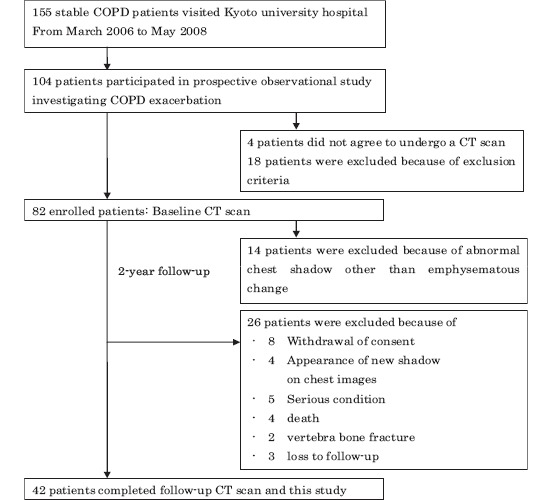
Patient disposition and reasons for exclusion. COPD, chronic obstructive pulmonary disease; CT, computed tomography.

The study protocol is summarized in Figure 1, and the details of patient disposition are provided in the result section. Briefly, from March 2006 to May 2008, we enrolled 82 of 104 patients with COPD who agreed to record exacerbations prospectively. The observation period was 2 years. Finally, follow-up CT scans were performed 2 years after study entry in 42 patients. The subject population in the present study was almost the same as in our previous study (15). The most notable difference between the two study populations was related to whether the patients receiving osteoporosis therapy were excluded. The ethics committee of Kyoto University approved the study (approval No. E182), and all patients provided their written informed consent to participate

## Exacerbation criteria and definition of stable condition

Exacerbations were defined as symptomatic deterioration requiring medical treatment (antibiotics and/or systemic corticosteroids) (15,19,20). Stable condition was defined as an exacerbation-free interval that lasted for more than 4 weeks. Symptomatic changes were assessed by a modified version of the East London cohort study criteria (7).

## Pulmonary function tests, CT acquisition, and calibration of CT numbers

Baseline and 2-year follow-up CT scans (Aquilion 64; Toshiba, Tokyo, Japan; slice thickness, 0.5 mm) and pulmonary function tests (Chestac-65V; Chest MI Corp, Tokyo, Japan) were performed under stable conditions. All pulmonary function tests and CT scans were performed after inhaling short-acting bronchodilators (salbutamol and ipratropium) as previously reported (5,21). CT numbers in the lung field were corrected using tracheal air density to prevent the X-ray tube aging effect (15,22).

## Analysis of low attenuation areas

To evaluate pulmonary emphysema quantitatively, the low attenuation area percentage (LAA%) was measured according to our previous reports (5,23).

## Measurement of BMD in vertebral bone

Using a modified method reported previously (5,17), BMD was measured in the thoracic vertebral bones (T4, T7, T10) derived from the CT scan density. In a previous report, BMD was calculated using one chest CT scan slice, but in the present study, it was calculated using five chest CT scan slices. Briefly, first a mid-vertebral slice was selected using reconstructed images (sagittal and coronal images) from 0.5-mm slice thickness CT images. Then, the elliptical region of interest (ROI) was encompassed manually as the largest possible area at the anterior portion of each vertebral body on five slices whose center was the chosen mid-vertebral slice.

Finally, the mean CT scan density of the ROI was measured. As in our previous report (5), BMD was calculated based on mean CT scan density using the formula (BMD (in milligrams per milliliter) = 0.767 × CT scan density (in HU) + 3.37). This calculating formula was validated in our previous report using calibration phantom which contained eight tubes of known concentration of hydroxyapatite (5). All images were measured by two pul-monologists (H.K. and N.T.) to minimize interobserver variability of the measurements, and their measurements were then averaged to obtain final values.

## Measurement of the change in BMD

The annual change in BMD (ABMD) was defined as follows:


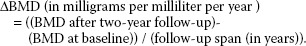


Annual BMD percentage change normalized with baseline BMD was adjusted as follows:


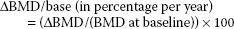


## Statistical analyses

All statistical analyses were performed using JMP 7 software (SAS Institute; Cary, NC). The data are expressed as medians (25^th^, 75^th^ percentile) unless otherwise indicated. The differences between groups were evaluated using the Mann-Whitney U-test. Data within groups were analyzed with the Wilcoxon signed-rank test. Relationships among data were assessed by Spearman's rank correlation test. To evaluate the relative contribution of exacerbations to the decrease in thoracic vertebral BMD, multivariate regression analysis was performed. A *p*-value less than 0.05 was considered significant.

## Results

### Patient characteristics

Baseline CT scans were performed on 82 patients who had participated in our prospective observational study investigating COPD (15,19,20). Of these patients, 14 were excluded because of abnormal chest shadows not associated with emphysematous changes seen on chest CT images at entry (Figure 1). Over the two-year follow-up, 26 patients were excluded for the following reasons: withdrawal of consent, appearance of a new shadow, serious condition, death, vertebral bone fracture, or lost to follow-up. Table 1 shows the characteristics of the study subjects (42 patients) who completed all of the follow-up examinations, including baseline and 2-year follow-up chest CT scans. During the observational period, 13 patients experienced exacerbations requiring treatment with antibiotics and/or systemic corticos-teroids at least once, and 29 patients experienced no exacerbations. Of 13 patients with a history of exacerbations, only 1 patient received systemic corticosteroid treatment (methylprednisolone 16 mg/day for 5 days). There were no significant differences in baseline clinical parameters, pulmonary function, CT parameters, or BMD between the groups (Table 2). Baseline pharmacological treatment including inhaled corticosteroids was not significantly different between the groups.

**Table 1:** Baseline characteristics of the study patients (N = 42)

Characteristics		Values
Age	70.0	(65.0,76.3)
Gender, male : female	39:	3
Height, meter	1.64	(1.60, 1.69)
Weight, kg	57.5	(53.5, 62.0)
Body mass index	21.0	(19.5, 23.3)
Smoking Status current : former	13:	39
Smoking history, pack-year	53.5	(42.5, 88.0)
FEV_1_, L	1.61	(1.12, 1.94)
%FEV_1_, %	56.4	(41.8, 69.4)
LAA% (-960), %	32.2	(25.0, 38.1)
D_LCO_ /V_A_, mL/min/mmHg/L	2.61	(1.84, 3.39)

Data are expressed as the median (25th, 75th percentile). FEV_1_, forced expiratory volume in 1 second; %FEV_1_, FEV_1_ % predicted; LAA%, percentage of low attenuation area; D_LCO_/V_A_, ratio of diffusing capacity to alveolar ventilation.

**Table 2:** Baseline characteristics of the two groups: Patients with and without a history of COPD exacerbations

	Exacerbation (−)		Exacerbation (+)		p-value
Subjects		29		13	
Exacerbations/year	0		0.50	(0.48, 0.83)	
Baseline characteristics					
Age, years	66.0	(62.5, 77.5)	72.0	(68.0, 74.0)	0.27
Gender, male:female	28 :	1	11 :	2	0.22
Body mass index	21.6	(19.9, 23.0)	20.1	(17.7, 24.3)	0.24
Smoking Status, current : former	11 :	18	2:	11	0.28
Smoking history, pack-year	57.0	(42.5, 99.0)	52.0	(41.5, 78.8)	0.57
FEV_1_, L	1.64	(1.12, 2.08)	1.45	(1.15, 1.91)	0.64
%FEV_1_, %	55.1	(39.3, 72.3)	61.0	(53.5, 66.9)	0.50
GOLD classifi					
stage I	4	(13.8)	1	(7.7)	
stage II	14	(48.3)	10	(76.9)	
stage III	10	(34.4)	1	(7.7)	
stage IV	1	(3.5)	1	(7.7)	
BMD, mg/ml					
LAA% (-960), %	31.8	(24.9, 38.1)	32.6	(24.9, 39.2)	0.87
D_LCO_/VA, mL/min/mmHg/L	2.68	(1.86, 3.36)	2.48	(1.43, 3.88)	0.59
PaO_2_, mmHg	78.9	(73.4, 84.2)	73.7	(70.7, 85.9)	0.64
ICS, Yes:No	4:	25	4 :	9	0.23
Tio, Yes:No	12 :	17	5 :	8	1.00

Data are expressed as the median (25th, 75th percentile). FEV_1_, forced expiratory volume in 1 second; %FEV_1_, FEV_1_ % predicted; GOLD, The Global Initiative for Chronic Obstructive Lung Disease; BMD, bone mineral density; LAA%, percentage of low attenuation area LAA%, percentage of low attenuation area; D_LCO_/V_A_, ratio of diffusing capacity to alveolar ventilation ICS, ihhaled corticosteroid; Tio, tiotropium.

## Impact of exacerbations on BMD, lung functions, and emphysematous change

**Figure 2:**
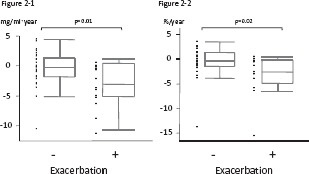
Annual change in thoracic vertebral BMD in patients without / with a history of exacerbations. **Figure 2-1** Annual absolute change in thoracic vertebral BMD in patients without / with a history of exacerbations (ΔBMD (mg/ml·year): −0.30 versus −3.78, p = 0.01). **Figure 2-2**. Annual percentage change in thoracic vertebral BMD in patients without/with a history of exacerbations based on baseline BMD. (ΔBMD/base (mg/ml·year): −0.60 versus −5.41, p = 0.02). The horizontal line is the median value, the box is the interquartile range, and the whiskers indicate the range, excluding outlying and extreme values (i.e., points with values ≥1.5 box lengths from the upper or lower limits of the box). BMD, bone mineral density; ΔBMD, annual change in thoracic BMD; ΔBMD/base, annual percentage change in BMD based on baseline BMD.

Changes in BMD, lung functions, emphysematous change, and PaO_2_ between patients with and without a history of exacerbations are shown in Table 3. In patients who experienced exacerbations, BMD and BMD/base decreased significantly after the follow-up period, whereas these parameters did not change in patients who experienced no exacerbations. The median annual changes in BMD and BMD/base were significantly greater in patients with than in those without exacerbations (ABMD mg/ml-year: −3.78 versus −0.30, p = 0.01, ABMD/base %: −5.41 versus −0.60, p = 0.02) (Table 2, Figure 2).

**Table 3:** Annual changes in thoracic vertebral BMD, lung function, and LAA%

	Exacerbation (−)		p-value (within group)	Exacerbation (+)		p -value (within group)	p-value (between groups)
BMD, mg/ml	−0.30	(−1.82, 1.33)	0.41	−3.78	(−5.81, −0.29)	0.002	0.01
BMD/base, %	−0.60	(−3.07, 2.63)	0.39	−5.41	(−10.6, −0.36)	0.002	0.02
BMI	0.00	(−0.53, 0.38)	0.65	−0.78	(−1.49, 0.70)	0.34	0.38
FEV_1_, mL	−51.4	(−117.0, −7.7)	0.0001	−75.0	(−97.9, −4.7)	0.0005	0.72
%FEV_1_, %	0.28	(−2.08,2.15)	0.74	0.00	(−1.72, 1.58)	0.91	0.85
D_LCO_ /V_A_, mL/min/mmHg/L	−0.13	(−0.32, 0.03)	0.01	−0.15	(−0.26, 0.03)	0.10	1.00
LAA% (−960), %	0.12	(−0.61, 1.27)	0.43	1.32	(−4.78, 1.31)	0.0005	0.01
PaO_2_, mmHg	−0.48	(−2.42, 1.80)	0.91	−1.00	(−4.78, 1.32)	0.20	0.29

Data are expressed as the median (25th, 75th percentile). BMD, bone mineral density in thoracic vertebrae ; BMD/base, percentage change in BMD compared with baseline BMD; FEV_1_, forced expiratory volume in 1 second; %FEV_1_, FEV_1_ % predicted D_LCO_ /V_A_, ratio of diffusing capacity to alveolar ventilation; LAA%, percentage of low attenuation area.

In agreement with our previous report (15), LAA% also increased significantly after the observation period in patients who experienced exacerbations (p = 0.01) (Table 3), whereas this parameter did not change in patients who experienced no exacerbations. There were no significant annual changes in pulmonary function test results such as FEV_1_ and D_LCO_ between the groups.

Table 4 shows the correlation between the annual change in BMD and associated factors for osteoporosis (exacerbation frequency and factors that have been shown to be associated with osteoporosis). The annual change in BMD was significantly correlated with the exacerbation frequency (Spearman's rank correlation coefficient ρ = -0.37, p = 0.02). On univariate analysis, baseline FEV_1_ was associated with the change in BMD (p = 0.04) whereas other factors such as BMI, smoking history, %FEV_1_ predict, and LAA% were not.

**Table 4:** Univariate associations with annual change in thoracic vertebral

Variables	Spearman's rank correlation (ρ)	p-value
Baseline characteristics		
Age	−0.18	0.26
Body mass index	−0.04	0.80
Smoking history	0.20	0.20
FEV_1_ absolute	0.32	0.04
%FEV1 predict	0.11	0.49
D_LCO_ /V_A_	0.16	0.30
LAA% (−960)	0.03	0.86
PaO_2_	0.29	0.07
Annual change		
Frequency of excerbation	−0.37	0.02
ΔFEV_1_	−0.16	0.32
Δ%FEV_1_	0.00	0.98
ΔBMI	0.05	0.75
ΔLAA%(−960)	−0.10	0.53
ΔPaO_2_	0.22	0.20

Relationships were assessed by Spearman's rank correlation test. BMD, bone mineral density; FEV_1_, forced expiratory volume in 1 second; %FEV_1_, FEV_1_ % predicted; D_LCO_ /V_A_, ratio of diffusing capacity to alveolar ventilation; LAA%, percentage of low attenuation area Δ means annual change in each variables.

On stepwise multivariate regression analysis (Table 5) to examine the relative contribution of each variable to predict the change in BMD, 5 factors that ranked in the top 5 on univariate analysis were selected as candidate explanatory variables (exacerbation, age, smoking index, FEVj, and PaO_2_). As a result, exacerbation and baseline PaO_2_ were included in this model as explanatory variables after stepwise variable selection (R^2^ = 0.20, p = 0.007, and R^2^ = 0.09, p = 0.03, respectively).

**Table 5:** Stepwise multivariate regression analysis showing the relative contribution of each variable to predict the change in BMD

	Coefficient	p-value	R^2^
Intercept	−0.094		
History of exacerbations, yes:no	−0.013	0.007	0.20
PaO_2_, mmHg	0.001	0.03	0.09
Cumulative R^2^			0.30

Exacerbation (two categories: the presence versus absence of a history of exacerbations), age, smoking history, baseline FEV_1_, and PaO_2_ were included as candidate independent variables. After stepwise variable selection, baseline factors (age, smoking history and FEV_1_) were excluded.

**Figure 3:**
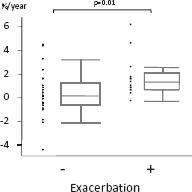
Annual emphysematous change represented as LAA% on CT in patients without / with a history of exacerbations. (LAA (%): 0.12 versus 1.32, p = 0.01) The horizontal line is the median value, the box is the interquartile range, and the whiskers indicate the range, excluding outlying and extreme values (i.e., points with values ≥1.5 box lengths from the upper or lower limits of the box). LAA, low attenuation area; CT, computed tomography.

## Discussion

In the present study, the analysis showed that a decrease in thoracic vertebral BMD was significantly associated with COPD exacerbations and baseline PaO_2_, while other factors known as risk factors for osteoporosis were not associated with the annual change in thoracic vertebral BMD. To the best of our knowledge, this is the first longitudinal study that investigated the impact of exacerbations on comorbidity in COPD patients.

In COPD patients, various risk factors, such as low BMI, smoking, age, inactivity, systemic inflammation, use of systemic corticosteroids (3), and LAA%, can be associated with osteoporosis (5). Although most of them are still controversial, only LAA% (5,6) and BMI (5,24) has been confirmed to correlate with BMD in cross-sectional studies. Osteoporosis is known as one of the common systemic manifestations in COPD (3), but the pathogenesis of osteoporosis in COPD patients has not been fully understood. Considering our result that the exacerbation frequency was significantly correlated with the decrease in thoracic vertebral BMD, the factors involved in exacerbations could be precipitating key factors and cause the adverse effect.

Of these factors, systemic inflammation is thought to play an important role in the progression of osteoporosis in COPD patients (3,25). In fact, tumor necrosis factor a (TNF-α) and interleukin-6 (IL-6) have been reported to be associated with osteoporosis in COPD patients (26), and both factors are known to stimulate osteoclasts and increase bone resorption through RANKL-mediated bone resorption *in vitro* (27). In patients with exacerbations, the levels of various pro-inflammatory markers, such as IL-6, were reported to be elevated (14). The elevated systemic inflammatory markers, especially TNF-α and IL-6, in patients with exacerbations might have caused the deterioration in BMD in the present study. Further study is necessary to explore these ideas.

It has been suggested that use of systemic steroids during exacerbations is one of the precipitating factors, but in the present study, only 1 of 13 patients with a history of exacerbations received systemic corticosteroid treatment. Even excluding this case, the study yielded the same conclusion. Thus, in clinical practice, it is important to pay attention to the patients who have a history of frequent exacerbations and treat them because they have a greater risk for osteoporosis progression, even if they do not receive systemic corticosteroids during exacerbations.

Multivariate analysis revealed that baseline PaO_2_ was associated with the BMD decline independently of a history of exacerbations. Although no studies have shown that hypoxemia is associated with osteoporosis, baseline hypoxemia may reflect the patients' dyspnea that causes inactivity, a risk factor for osteoporosis. Alternately, hypoxemia itself has adverse effects on osteoporosis in COPD patients (28). To confirm this finding and causal association, information about dyspnea and activity should be assessed in a future study.

We performed a preliminary study of the effect of osteoporosis therapy on the change in thoracic vertebral BMD. To explore the effect of osteoporosis therapy on BMD, we measured the change in BMD in 18 patients with a history of exacerbations using the same method as in the present study (online supplement e-Table 1). Osteoporosis therapy significantly improved the decrease in BMD compared to no osteoporosis therapy in patients with a history of exacerbations (p = 0.002, online supplement e-Table 2). These results suggest the potential of osteoporosis therapy, especially in patients with a history of exacerbations. This finding should be investigated in a future study.

This study had several limitations. First, the sample size was small. However, this was a single center study using only one scanner, so that the instability of CT scanners was less problematic than in multicenter studies.

In addition, exacerbations were prospectively recorded by at least two respiratory physicians who were unaware of CT data. These advantages were thought sufficient to overcome the small sample size. Moreover, one large observational study, the Evaluation of COPD Longitudinally to Identify Predictive Surrogate End-points study (ECLIPSE study), showed that the co-existence of osteoporosis was associated with the occurrence of exacerbations in a cross-sectional cohort (29). However, they did not examine the influence of exacerbations on osteoporosis progression. The findings in the present longitudinal study further deepen our understanding of the relationship between COPD exacerbations and osteoporosis progression that ECLIPSE suggested. In the present study we could detect the progression of LAA in those who have a history of exacerbations, but couldn't detect the decline in FEV_1_. It is partly because this study was small sample study.

Second, BMD in the present study was calculated on the chest CT data using our formula that was validated in our previous report (5). The data of the present study did not include Dual Energy X-ray Absorptiometry (DEXA), which is the standard method to estimate BMD. The lack of DEXA data could be a limitation, but it has been reported that CT scan density showed a highly significant positive correlation with pathological measurements of vertebral bone density (30). As described in the introduction section BMD of the vertebra assessed by chest CT scan is clinically useful to predict vertebral compression fractures that could deteriorate pulmonary functions. Moreover, chest CT scan can detect emphy-sematous change (LAA) and BMD simultaneously without additional X-ray exposure by DEXA.

Third the data of the present study subjects lack some confounding factors that could affect BMD, for example activity, diet intake (calcium and vitamin D intake), and loss of fat-free mass, since it has been suggested that inactivity could cause osteoporosis in COPD patients (31). The present study did not include some markers of bone metabolism (for example values for 25-hydroxyvi-tamin D) and systemic inflammation either. Future research is needed to assess the effect of other factors that were lacking in the present study and to clarify the mechanism of osteoporosis progression with COPD exacerbations.

## Conclusions

In conclusion, the present study demonstrated that the decrease in thoracic vertebral BMD was greater in patients with a history of exacerbations than in those without a history of exacerbations. These data suggest that osteoporosis progression should be evaluated in COPD patients, especially in those with a history of frequent exacerbations.

## Abbreviations

BMD: bone mineral density
BMI: body mass index
COPD: chronic obstructive pulmonary disease
CT: computed tomography
D_LCO_: diffusion capacity of carbon monoxide
ECLIPSE: Evaluation of COPD Longitudinally to Identify Predictive Surrogate End-points study (ECLIPSE study)
FEV_1_: forced expiratory volume in 1 second
GOLD: the Global Initiative for Chronic Obstructive Lung Disease
ICS: inhaled corticosteroid
IL-6: interleukin-6
LAA: low attenuation area
PaO_2_: partial pressure of oxygen in arterial blood
RANKL: receptor activator of nuclear factor kappa-B ligand
ROI: region of interest
TNF-α: tumor necrosis factor a
V_A_: alveolar ventilation
VCF: vertebral compression fracture.
